# Mixing Matrix-corrected Whole-body Pharmacokinetic Modeling Using Longitudinal Micro-computed Tomography and Fluorescence-mediated Tomography

**DOI:** 10.1007/s11307-021-01623-y

**Published:** 2021-07-06

**Authors:** Simin Zuo, Wa’el Al Rawashdeh, Stefanie Rosenhain, Zuzanna Magnuska, Yamoah Grace Gyamfuah, Fabian Kiessling, Felix Gremse

**Affiliations:** 1 Institute for Experimental Molecular Imaging, Helmholtz-Institute for Biomedical Engineering, RWTH Aachen University Clinic, 52074 Aachen, Germany; 2 Miltenyi Biotec GmbH, 51429 Bergisch Gladbach, Germany; 3 Comprehensive Diagnostic Center Aachen (CDCA), 52074 Aachen, Germany; 4 Fraunhofer Institute for Digital Medicine MEVIS, 28359 Bremen, Germany; 5 Gremse-IT GmbH, Dennewartstr. 25, 52068 Aachen, Germany

**Keywords:** Mixing matrix, Pharmacokinetic modeling, Fluorescence-mediated tomography, Computed tomography, Intensity diffusion, Relative blood volume, Elimination routes, Retention sites

## Abstract

**Purpose:**

Pharmacokinetic modeling can be applied to quantify the kinetics of fluorescently labeled compounds using longitudinal micro-computed tomography and fluorescence-mediated tomography (μCT-FMT). However, fluorescence blurring from neighboring organs or tissues and the vasculature within tissues impede the accuracy in the estimation of kinetic parameters. Contributions of elimination and retention activities of fluorescent probes inside the kidneys and liver can be hard to distinguish by a kinetic model. This study proposes a deconvolution approach using a mixing matrix to model fluorescence contributions to improve whole-body pharmacokinetic modeling.

**Procedures:**

In the kinetic model, a mixing matrix was applied to unmix the fluorescence blurring from neighboring tissues and blood vessels and unmix the fluorescence contributions of elimination and retention in the kidney and liver compartments. Accordingly, the kinetic parameters of the hepatobiliary and renal elimination routes and five major retention sites (the kidneys, liver, bone, spleen, and lung) were investigated in simulations and in an *in vivo* study. In the latter, the pharmacokinetics of four fluorescently labeled compounds (indocyanine green (ICG), HITC-iodide-microbubbles (MB), Cy7-nanogels (NG), and OsteoSense 750 EX (OS)) were evaluated in BALB/c nude mice.

**Results:**

In the simulations, the corrected modeling resulted in lower relative errors and stronger linear relationships (slopes close to 1) between the estimated and simulated parameters, compared to the uncorrected modeling. For the *in vivo* study, MB and NG showed significantly higher hepatic retention rates (P<0.05 and P<0.05, respectively), while OS had smaller renal and hepatic retention rates (P<0.01 and P<0.01, respectively). Additionally, the bone retention rate of OS was significantly higher (P<0.01).

**Conclusions:**

The mixing matrix correction improves pharmacokinetic modeling and thus enables a more accurate assessment of the biodistribution of fluorescently labeled pharmaceuticals by μCT-FMT.

## Introduction

Fluorescence-mediated tomography (FMT) is an imaging technique used to assess the three-dimensional distribution of fluorescent probes in preclinical studies [1–3]. In recent years, the sensitivity and accuracy of FMT have notably increased, resulting in improved detection of fluorescent probes in deep tissue regions [4–7]. Therefore, FMT is a promising modality to quantify the whole-body biodistribution of probes in mice [8–11]. In this context, the application of FMT can also help reduce the number of mice required for biomedical and histological examinations in longitudinal studies.

One main limitation of the FMT technique is the lack of accurate and sharp localization of fluorescently labeled compounds *in vivo*, especially in deep tissue regions [12, 13]. In previous studies, combining FMT with a high-resolution anatomical imaging modality, such as micro-computed tomography (μCT), could substantially enhance its diagnostic quality [12, 14–16]. The shape information and organ-specific absorption and scattering maps derived from the μCT scans can be incorporated into FMT reconstruction algorithms, thus improving the quantification and localization of fluorescence signals [6, 7, 14, 17, 18]. Additionally, the organ-specific pharmacokinetic profiles can be extracted by fusing the FMT images and μCT-based organ segmentation [17–19].

Pharmacokinetic modeling is used to quantify kinetic behaviors from pharmacokinetic curves [20–25]. Compartmental modeling has been routinely applied to derive kinetic parameters in previous studies, facilitating a better understanding of the information contained in the dynamic *in vivo* imaging data [26]. In contrast to high-resolution imaging methods like CT and ultrasound, in FMT, the fluorescence is blurred between adjacent organs and tissues due to the lower resolution. Fluorescence of small imaging voxels comparable to the finite spatial resolution of FMT (1–3 mm) influences each other between neighboring tissues [3, 8, 27, 28]. The limitations of the reconstruction for this ill-posed problem in FMT also cause blurriness, which could be remarkably improved by the incorporation of CT scans [6].

Additionally, vasculature inside organs also affects the fluorescence measurements of tissue compartments [8]. Furthermore, fluorescence data obtained from the liver and kidneys do not just represent retention kinetics of probes but also contain dynamic information about probe elimination—the liver, for example, consists of hepatocytes for elimination activities and Kupffer cells as retention sites [29–31]. In pharmacokinetic modeling, the fluorescence contributions of elimination and retention in the kidney and liver compartments should be considered. To reduce these fluorescence blurring and mixing effects, we introduce a mixing matrix into the pharmacokinetic modeling to deconvolute the underlying accurate parameters.

Thus, this study aimed to improve parameter estimation in pharmacokinetic modeling using longitudinal μCT-FMT data. For the first time, the mixing matrix was applied to unmix the fluorescence blurring from neighboring tissues and blood vessels and differentiate the elimination and retention sub-compartments in the liver and kidneys. The relevance of the mixing matrix was assessed in simulations and comparatively analyzed using *in vivo* data.

## Materials and Methods

### *In Vivo* μCT-FMT Imaging

All animal experimental procedures were approved by the Governmental Review Committee on Animal Care. Healthy BALB/c nude mice (Charles River Laboratory, Sulzfeld, Germany) were anesthetized to be imaged by hybrid μCT-FMT (μCT: Tomoscope 30s Duo, CT Imaging GmbH, Erlangen, Germany; FMT: FMT 2500, PerkinElmer, Waltham, MA, USA), at 0.25, 2, 4, 8, 24, 48, and 72 h after intravenous injection.

In the animal experiments, four fluorescently labeled compounds (five female mice per group, 2 nmol in 100 μl solution per mouse)—indocyanine green (ICG, with a molecular weight of 774.96 g/mol), HITC-iodide-microbubbles (MB, with a diameter of 2–3 μm), Cy7-nanogels (NG, with a diameter of 117 ± 5 nm), and OsteoSense 750 EX (OS, with a molecular weight of 1101.1 g/mol)—were chosen to cover various elimination routes and retention sites, as described in detail elsewhere [18]. These fluorescent compounds have well-known but different pharmacokinetic properties. After intravenous administration, ICG is almost exclusively eliminated by the liver with low non-specific retention in cells and tissues. MB and NG are mainly taken up by the Kupffer cells and biodegraded by the liver. The bone-targeting OS is mainly eliminated by the kidneys. All of them are water-soluble and can be fluorescently excited by the 745-nm FMT channel.

**Fig. 1.**
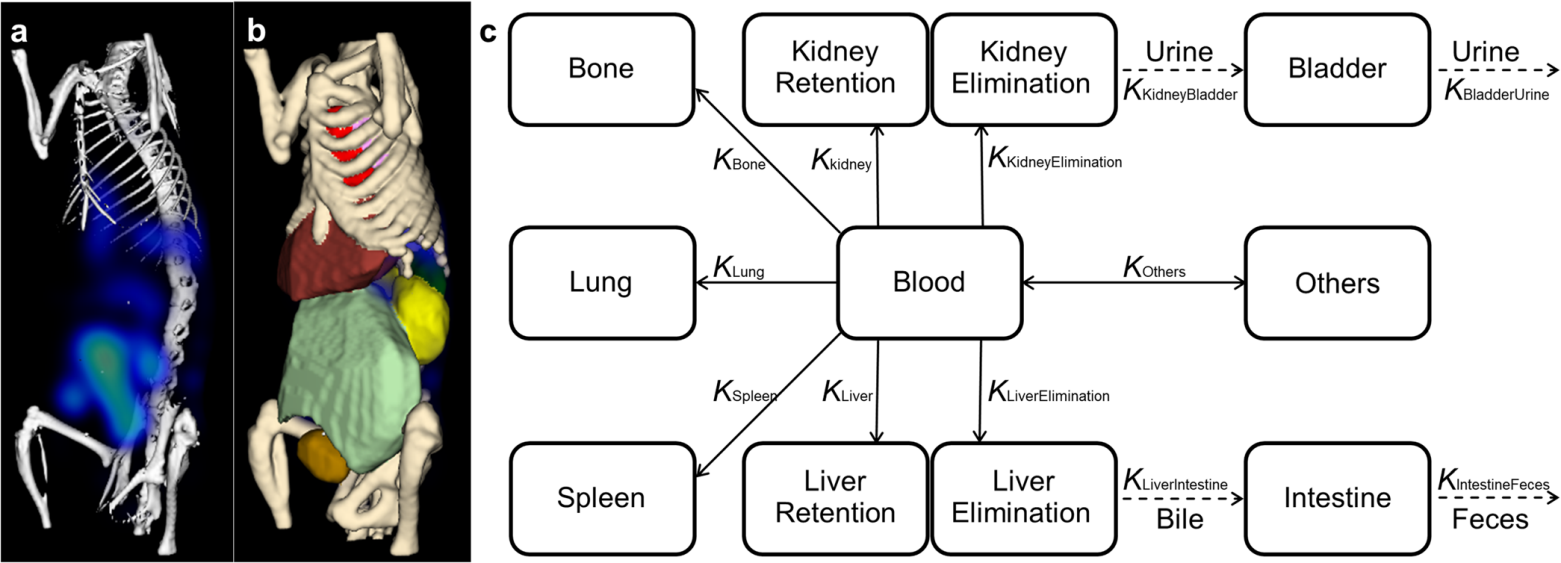
Whole-body pharmacokinetic modeling using μCT-FMT. **a** Fluorescence reconstruction with μCT information, **b** combined with μCT-based organ segmentation (heart, red; kidney, yellow; bladder, gold; liver, dark red; intestine, light green; bone, beige; spleen, green; lung, pink). **c** Simplified whole-body pharmacokinetic model. The blood flow (blood) facilitates compound distribution to major organs (liver, kidneys, spleen, bone, and lung). Compounds could retain in major organs and are usually eliminated by the liver or kidneys. Corresponding kinetic rates (retention rates and elimination rates) are represented by kinetic parameters (***K***). Eight unknown kinetic parameters (solid arrows) are iteratively estimated by pharmacokinetic modeling. Four known parameters (dashed arrows) were derived from animal experiment data (from Al Rawashdeh and Zuo et al. [18]).

The 3D fluorescence images were obtained by FMT reconstruction, which uses μCT information acquired for each mouse and time point (Fig. 1a) [6]. The reconstruction method “corrected” the fluorescence underestimation caused by a rich blood supply in some major organs, such as the liver and kidneys [6]. Based on the fused μCT and 3D fluorescence image data, the fluorescence biodistribution data (from 7 time points) of 8 organs (the heart, kidneys, bladder, liver, intestine, bone, spleen, and lung) was extracted using μCT-based organ segmentation (Fig. 1b) [18].

### Pharmacokinetic Modeling

Our simplified whole-body pharmacokinetic model describes the exchange of fluorescently labeled compounds between compartments [18]. This model was carefully designed to balance the number of unknown parameters and the measurements, thus achieving global convergence in simulations.

The whole-body pharmacokinetic model consists of two main elimination routes and five major retention sites. To be specific, several features are incorporated to make the model physiologically realistic and applicable in this study using intravenous injection (Fig. 1c):

(i) The blood compartment is the driving force of compound distribution (blood) [18].
(ii) Compounds are assumed to irreversibly retain in five major compartments (liver, kidney, spleen, bone, and lung) [29, 32].
(iii) Compounds are eliminated by the hepatic and/or renal elimination routes (liver, intestine, kidney, and bladder) [29].
(iv) Compounds can diffuse into the rest of the body and back into the blood (others).

Without loss of generality, the mass-balancing formulation in the context of pharmacokinetic modeling could be described as:

1 $$ \frac{d\boldsymbol{I}}{dt}=\boldsymbol{KI} $$

where vector ***I*** represents the amount of fluorescently labeled compounds in compartments, and ***K*** is the sparse adjacency matrix representing the multi-parameter set, consisting of eight unknown kinetic parameters. The unknown parameters are calculated by fitting the model to the longitudinal μCT-FMT data. A constraint 0.001 < ***K***_***ij***_ < 0.02 is implemented in parameter estimation to ensure a physiologically meaningful range of kinetic parameters.

### Mixing Matrix Correction

The fluorescence measurements in segmented organs obtained from hybrid μCT-FMT are not exclusively the fluorescence intensity inside the corresponding compartments. In this study, we introduce a mixing matrix (***M***) to “correct” the measurement of selected organs (***I***_***m***_) and compute the fluorescence intensity in compartments (***I***):

2 $$ {\boldsymbol{I}}_{\boldsymbol{m}}=\boldsymbol{MI} $$

where ***M*** is a sum of three mixing terms:

(i) fluorescence blurring between adjacent compartments,
(ii) fluorescence blurring from regional blood vessels, and
(iii) fluorescence fusion of the elimination and retention activities of compounds inside the kidneys/liver.

The first mixing term, the fluorescence blurring from neighboring tissues due to photon diffusion and the limited spatial resolution of the FMT device, is termed “intensity diffusion” [28, 33]. To obtain the matrix of “intensity diffusion,” a three-dimensional Gaussian filter (FWHM: 0.5 mm) was applied to the mask of each organ segmentation. Then, the intensity average is computed in the other organ mask to obtain the corresponding “intensity diffusion” value (IDV). The IDV matrix was obtained using the hybrid μCT-FMT data:

$$ \mathrm{IDV}=\left[\begin{array}{ccc}\begin{array}{ccc}0.717& 0.022& 0.023\\ {}0.039& 0.818& 0.013\\ {}0.007& 0.002& 0.872\end{array}& \begin{array}{ccc}0.122& 0.000& 0.000\\ {}0.043& 0.000& 0.000\\ {}0.022& 0.000& 0.013\end{array}& \begin{array}{ccc}0.000& 0.000& 0.115\\ {}0.000& 0.000& 0.086\\ {}0.002& 0.000& 0.071\end{array}\\ {}\begin{array}{ccc}0.019& 0.004& 0.012\\ {}0.000& 0.000& 0.002\\ {}0.000& 0.000& 0.008\end{array}& \begin{array}{ccc}0.682& 0.001& 0.004\\ {}0.044& 0.650& 0.000\\ {}0.004& 0.000& 0.898\end{array}& \begin{array}{ccc}0.000& 0.000& 0.248\\ {}0.044& 0.000& 0.252\\ {}0.007& 0.000& 0.067\end{array}\\ {}\begin{array}{ccc}0.000& 0.000& 0.009\\ {}0.000& 0.000& 0.000\\ {}0.005& 0.002& 0.011\end{array}& \begin{array}{ccc}0.002& 0.005& 0.046\\ {}0.005& 0.000& 0.001\\ {}0.074& 0.001& 0.018\end{array}& \begin{array}{ccc}0.806& 0.000& 0.129\\ {}0.000& 0.742& 0.249\\ {}0.005& 0.002& 0.785\end{array}\end{array}\right], $$

where each IDV represents the fluorescence contribution from adjacent compartments (row: lung, blood, liver, bone, spleen, intestine, kidneys, bladder, and others) to the measured organs (column: lung, blood, liver, bone, spleen, intestine, kidneys, bladder, and others).

The second mixing term represents the fluorescence blurring from vasculature inside the organs, determined by the relative blood volume of organs (rBV). The rBV data were obtained from previous studies [34].

The third mixing term incorporates the elimination and retention sub-compartments in the liver and kidneys (Fig. 1c). The mixing matrix involving these three mixing terms was included in the forward model and the iterative model fitting to deconvolute the kinetic parameters.

### Kinetic Parameter Estimation

With the mixing matrix-corrected forward model, an iterative strategy based on the kinetic model was applied to estimate the best-fit kinetic parameters.

(i) Mixing Matrix-Corrected Kinetic Modeling

**Fig. 2.**
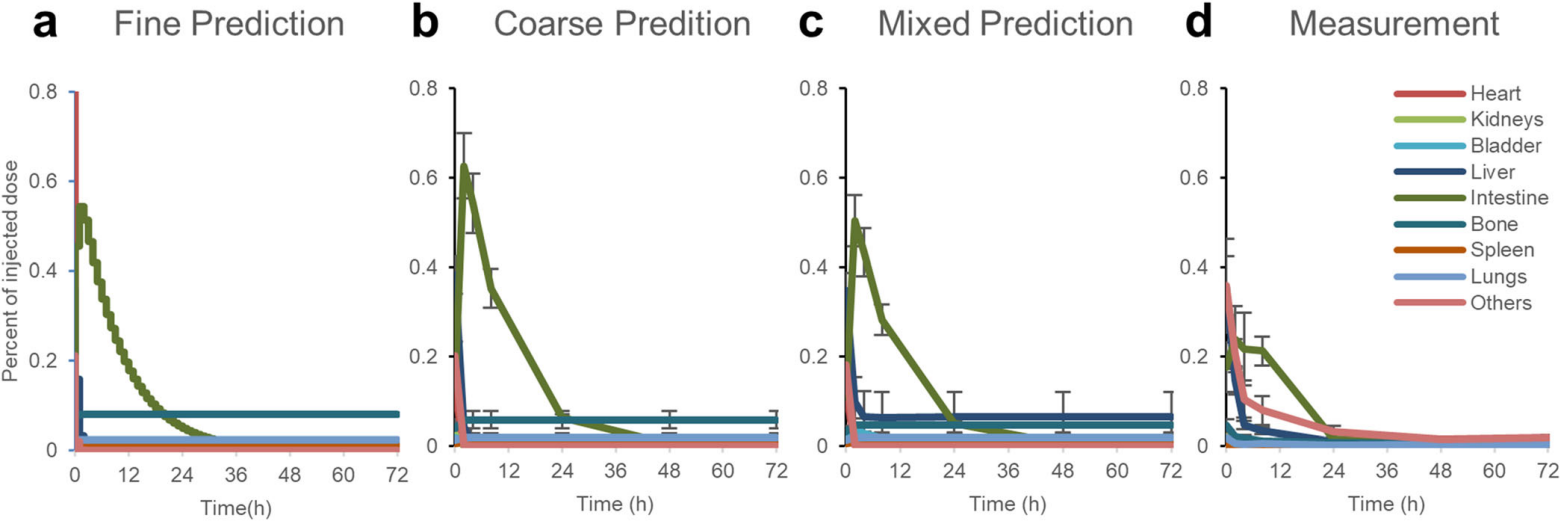
Modeling steps of the pharmacokinetic curves. **a** Fine-grained (high temporal resolution) prediction ${\hat{\boldsymbol{I}}}_{\boldsymbol{f}}$ is generated based on the pharmacokinetic model. **b** Coarse prediction ${\hat{\boldsymbol{I}}}_{\boldsymbol{c}}$ is then extracted from the fine curves at sparse time points 0.25, 2, 4, 8, 24, 48, and 72 h after i.v. injection. **c** Mixed prediction ${\hat{\boldsymbol{I}}}_{\boldsymbol{m}}$ is calculated by multiplying the coarse prediction ${\hat{\boldsymbol{I}}}_{\boldsymbol{c}}$ using the mixing matrix. **d** The measurement ***I***_***m***_ is from animal experiment data using hybrid μCT-FMT (ICG) [18].

A fine-grained prediction ${\hat{\boldsymbol{I}}}_{\boldsymbol{f}}$ (Fig. 2a) is computed by applying the 4th-order Runge-Kutta algorithm to the differential equation (Eq. 1):

3 $$ \frac{d{\hat{\boldsymbol{I}}}_{\boldsymbol{f}}}{dt}=\boldsymbol{K}{\hat{\boldsymbol{I}}}_{\boldsymbol{f}}. $$

This prediction is computed at a sufficiently high temporal resolution to avoid numerical errors stemming from using coarse steps in the Runge-Kutta method. The fine prediction ${\hat{\boldsymbol{I}}}_{\boldsymbol{f}}$ is then coarsely sampled to obtain the coarse prediction ${\hat{\boldsymbol{I}}}_{\boldsymbol{c}}$ (Fig. 2b) at defined time points, such as the 7 time points at 0.25, 2, 4, 8, 24, 48, and 72 h in the *in vivo* experiments. ${\hat{\boldsymbol{I}}}_{\boldsymbol{c}}$ is further modified by the mixing matrix ***M*** to generate the mixed prediction ${\hat{\boldsymbol{I}}}_{\boldsymbol{m}}$—the prediction of measurement in organs (Fig. 2c):

4 $$ {\hat{\boldsymbol{I}}}_{\boldsymbol{m}}=\boldsymbol{M}{\hat{\boldsymbol{I}}}_{\boldsymbol{c}} $$

(ii) Cost Function-Based Parameter Estimation

To estimate the best-fit ***K***, the cost function is defined as:

5 $$ \boldsymbol{f}\left(\boldsymbol{K}\right)=\sum {\left({\hat{\boldsymbol{I}}}_{\boldsymbol{m}}-{\boldsymbol{I}}_{\boldsymbol{m}}\right)}^2+\boldsymbol{c}{\boldsymbol{K}}^{\mathbf{2}} $$

In this model fitting problem, the first part $\sum {\left({\hat{\boldsymbol{I}}}_{\boldsymbol{m}}-{\boldsymbol{I}}_{\boldsymbol{m}}\right)}^2$ represents the sum-squared error between the prediction ${\hat{\boldsymbol{I}}}_{\boldsymbol{m}}$ (Fig. 2c) and measurement ***I***_***m***_ (Fig. 2d). The regularization term ***cK***^**2**^ is a parameter penalty to avoid extremely large solutions of ***K***.

Then, the nonlinear conjugate gradient method is used to minimize the penalized cost function ***f***(***K***). Iteratively, gradients are computed using adjoint algorithmic differentiation to determine a descent direction and perform a line search [35]. Accordingly, the eight unknown kinetic parameters are estimated by iteratively conducting these two steps.

### Simulations

Numerical simulations were performed to assess whether kinetic parameters can be deconvoluted using this model and to estimate the effect of the mixing matrix. One hundred groups of kinetic parameters were randomly generated, independently and uniformly distributed in the range 0.001 to 0.02. Fine measurements ${\hat{\boldsymbol{I}}}_{\boldsymbol{f}}$ were then simulated based on the proposed kinetic modeling. Coarsely sampled measurements ${\hat{\boldsymbol{I}}}_{\boldsymbol{c}}$ were extracted from the fine measurements ${\hat{\boldsymbol{I}}}_{\boldsymbol{f}}$ at 0.25, 2, 4, 8, 24, 48, and 72 h. Besides, a 10 % relative noise was randomly added to the sampled measurements. With the noisy measurements, the mixing matrix-corrected model was applied to estimate the kinetic parameters.

The performance of the kinetic parameter estimation with mixing matrix correction was studied and compared to the estimation without correction. The feasibility and reliability of the corrected modeling were evaluated in terms of the linear relationships of parameters and the relative errors of the estimated parameters compared to the uncorrected modeling.

To investigate the dependency of the corrected modeling on sampling density, different temporal sampling schemes were applied. More densely sampled measurements ${\hat{\boldsymbol{I}}}_{\boldsymbol{c}}$ (at 7, 20, and 50 time points) were extracted from the fine measurements ${\hat{\boldsymbol{I}}}_{\boldsymbol{f}}$. Furthermore, the corrected modeling with more early sampling points at the pharmacokinetic process, such as 7-early, i.e., seven measurements at 0, 0.25, 0.5, 2, 12, 24, and 48 h, more densely sampled in the first hour, was investigated to explore the influence of data sampling during the early phase of probe distribution.

### Statistical Analysis

In simulations, linear regression analysis was used to examine the linear relationship and the goodness of fit between the randomly chosen and estimated parameters. In the *in vivo* study, the kinetic parameters estimated by the uncorrected and mixing matrix-corrected modeling were compared using a paired two-tailed t-test.

## Results

### Simulations

Mathematical simulations were designed to evaluate if the mixing matrix-corrected model could recover the kinetic parameters and to assess the effect of the mixing matrix on the parameter estimation.

**Fig. 3.**
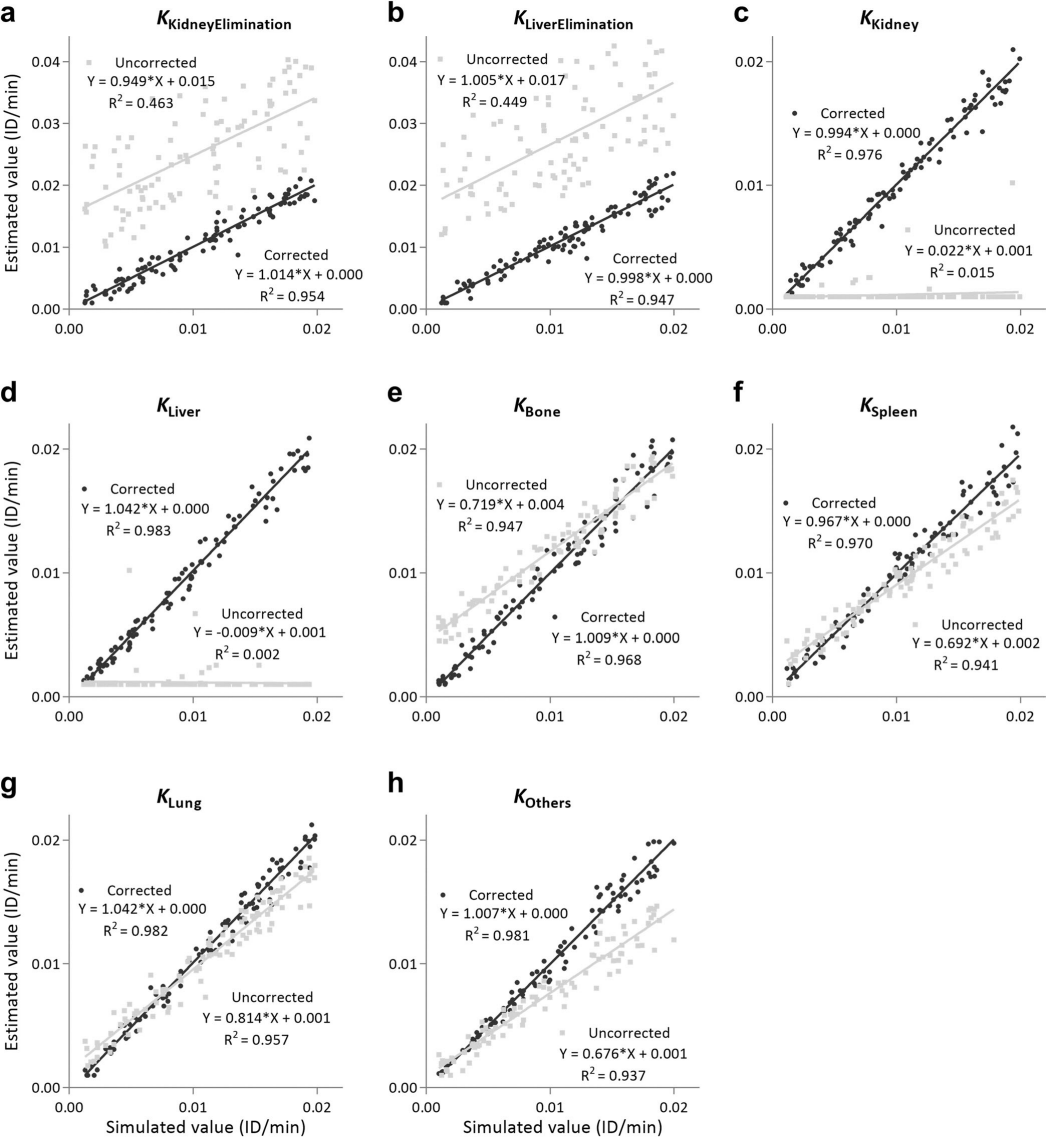
Comparison of estimated parameters using the uncorrected and mixing matrix-corrected kinetic modeling in simulations. Measurements were simulated using the mixing matrix, and random noise was added. Only the estimates using the mixing matrix have strong linear relationships with the original parameters (slopes and R-squared close to 1) for all parameters. **a**, **b** Without the mixing matrix, the kidney and liver’s elimination rates, the estimates have higher variances (small R-squared values) and a higher values bias. **c**, **d** Without the mixing matrix, the renal and hepatic retention rates cannot be recovered. **e**–**h** The other estimated parameters (bone, spleen, lung, and others) with uncorrected modeling have less linear fit lines.

**Fig. 4.**
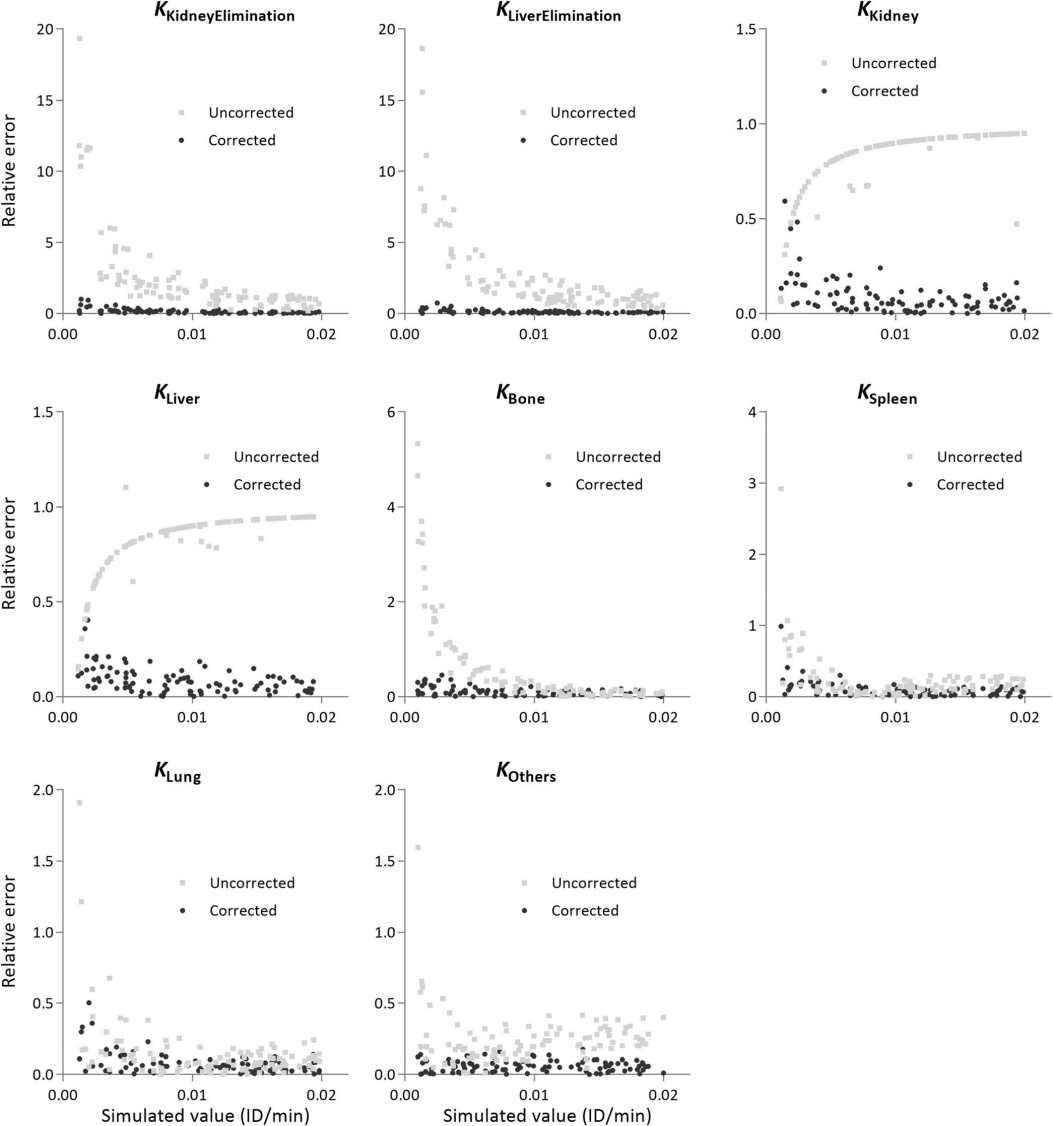
Relative errors of estimated parameters using the uncorrected and mixing matrix-corrected kinetic modeling in simulations. Measurements were simulated using the mixing matrix, and random noise was added. The relative errors of estimated parameters are notably smaller using mixing matrix correction, especially for the kidney and liver’s elimination and retention rates. Smaller relative errors are achieved with parameters larger than 0.005. Simulated parameters are randomly generated, to obtain simulated measurements.

Figure 3 shows the comparison of kinetic parameters estimated by the corrected and uncorrected modeling. With mixing matrix correction, all eight estimated parameters are approximately linear related to simulated parameters (slopes close to 1 and high R-squared values). In contrast, the parameters estimated by the uncorrected model have higher variances and are less linear, especially the hepatic and renal retention parameters. For example, the slope of the hepatic retention rate (*K*_Liver_) is 1.042 ± 0.014 with correction, whereas the uncorrected modeling is −0.009 ± 0.018 (Fig. 4). The strong linear relationship between the estimated and simulated parameters confirmed that the mixing matrix correction could significantly improve the parameter estimation in whole-body pharmacokinetic modeling.

The relative errors of kinetic parameters were also compared. Figure 4 shows the error distribution of the estimated parameters using the corrected and uncorrected modeling. With mixing matrix correction, estimated parameters have smaller relative errors, especially for the kidney and liver’s elimination and retention rates. Despite 10 % noise added to the measurements, the kinetic parameters estimated by the corrected modeling only have an average relative error of 9.77 %, compared to the average relative error of 98.41 % by the uncorrected modeling. Additionally, we found that larger simulated parameters (>0.005) could result in smaller relative errors in parameter estimation, which is reasonable because small parameter changes are hard to estimate under the presence of noise.

To assess the dependency of parameter estimation on sampling density, we investigated the effect of different temporal sampling schemes on parameter estimation using mixing matrix-corrected modeling.

Table 1 shows that sampling with more data points reduces the relative errors of estimated parameters. Moreover, the same amounts of measurements with more early samples in the pharmacokinetics (especially within the first hour) can suppress much more noise in parameter estimation, such as the “7-early” sampling scheme with 5.76 % averaged relative errors compared to the “7” sampling scheme with 9.77 %. The noise reduction is reasonable because of the fast exchange of compounds between compartments in the early phase of probe distribution.

**Table 1.** Relative errors of kinetic parameters with six temporal sampling schemes using the mixing matrix-corrected modeling. 7-early means 7 measurements with more dense sampling in the early pharmacokinetics (especially within the first hour), 20-early and 50-early in a similar way. A 10 % relative noise is randomly added to the simulated measurements

	Relative error					
Temporal sampling	7	20	50	7-early	20-early	50-early
*K*_KidneyElimination_	14.9 %	13.9 %	12.5 %	10.3 %	6.37 %	4.88 %
*K*_LiverElimination_	12.2 %	12.8 %	11.4 %	6.60 %	4.78 %	4.62 %
*K*_Kidney_	8.65 %	5.99 %	5.23 %	4.26 %	3.83 %	3.72 %
*K*_Liver_	8.69 %	6.19 %	5.58 %	5.29 %	3.89 %	3.66 %
*K*_Bone_	10.6 %	7.74 %	6.01 %	5.41 %	3.84 %	3.57 %
*K*_Spleen_	9.49 %	7.32 %	5.79 %	4.65 %	3.97 %	3.78 %
*K*_Lung_	7.50 %	6.08 %	5.05 %	4.40 %	3.82 %	3.58 %
*K*_Others_	6.14 %	6.44 %	6.48 %	5.17 %	5.29 %	5.14 %
Average relative error	9.77 %	8.31 %	7.26 %	5.76 %	4.47 %	4.12 %

### *In Vivo* Study

The kinetic parameters of four fluorescently labeled compounds were estimated using the mixing matrix-corrected modeling and the uncorrected one using longitudinal μCT-FMT imaging data. The kinetic modeling consists of seven kinetic parameters consisting of two elimination routes and five retention sites.

#### Elimination

**Fig. 5.**
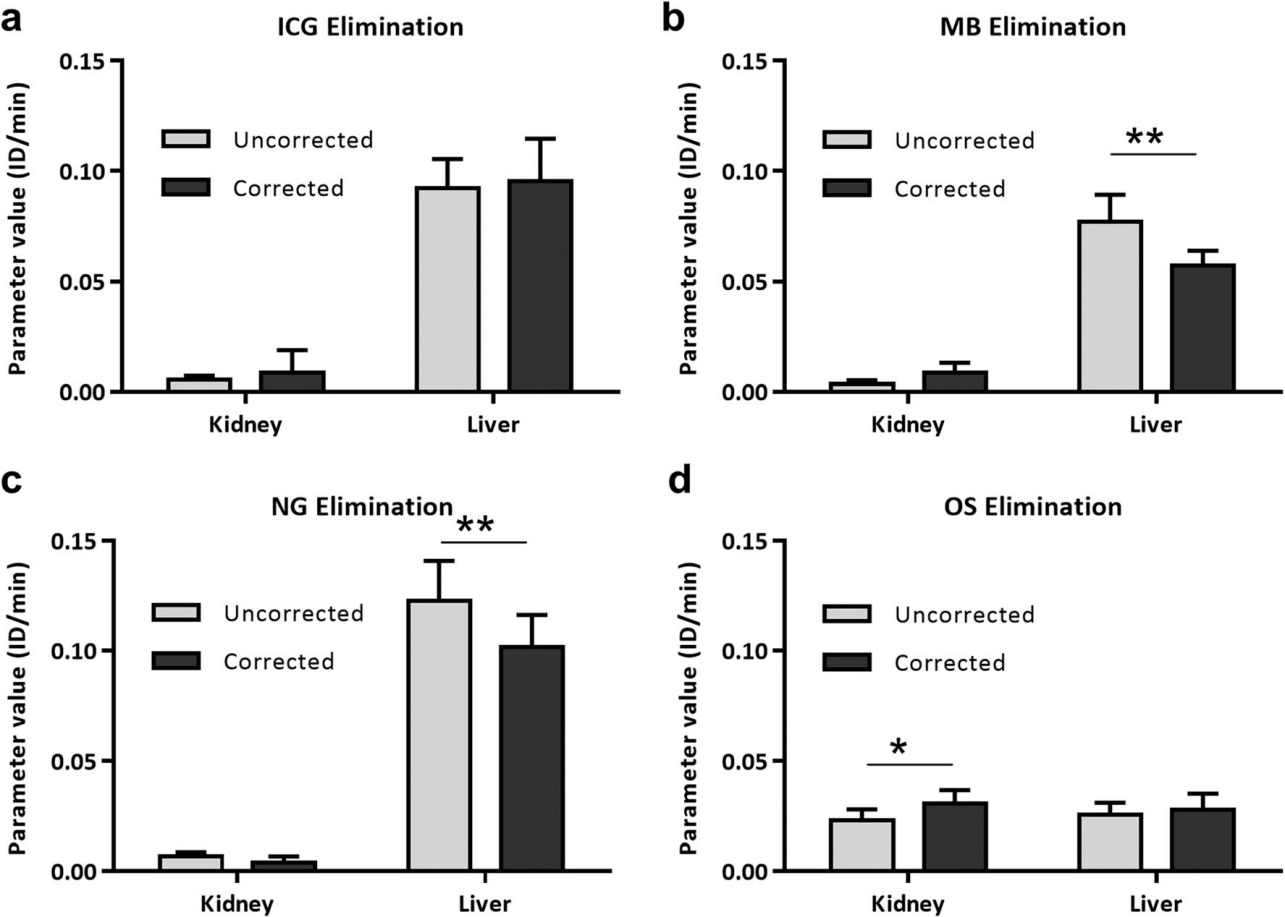
Hepatic and renal elimination of ICG, MB, NG, and OS using the uncorrected and mixing matrix-corrected modeling. **a**–**c** ICG, MB, and NG are mainly eliminated by the liver. **d** With correction, the renal elimination rate of OS was slightly higher compared to the rates without the correction (P<0.05). **b**, **c** MB and NG’s hepatic elimination rates are smaller using correction (P<0.01 and P<0.01, respectively). The paired two-tailed t-test was used in the comparison of elimination rates between the corrected and uncorrected modeling using μCT-FMT data, n=5.

Figure 5 shows that ICG, MB, and NG are mainly eliminated by the liver, whereas OS is eliminated by the liver and kidneys. It was noted that using the mixing matrix correction, the hepatic elimination rate of ICG and the renal elimination rate of OS were slightly larger, compared to the rates without the correction (OS: P<0.05) (Fig. 5a, d). However, the hepatic elimination rates of MB and NG were significantly smaller using the mixing matrix (P<0.01 and P<0.01, respectively) (Fig. 5b, c).

#### Retention

**Fig. 6.**
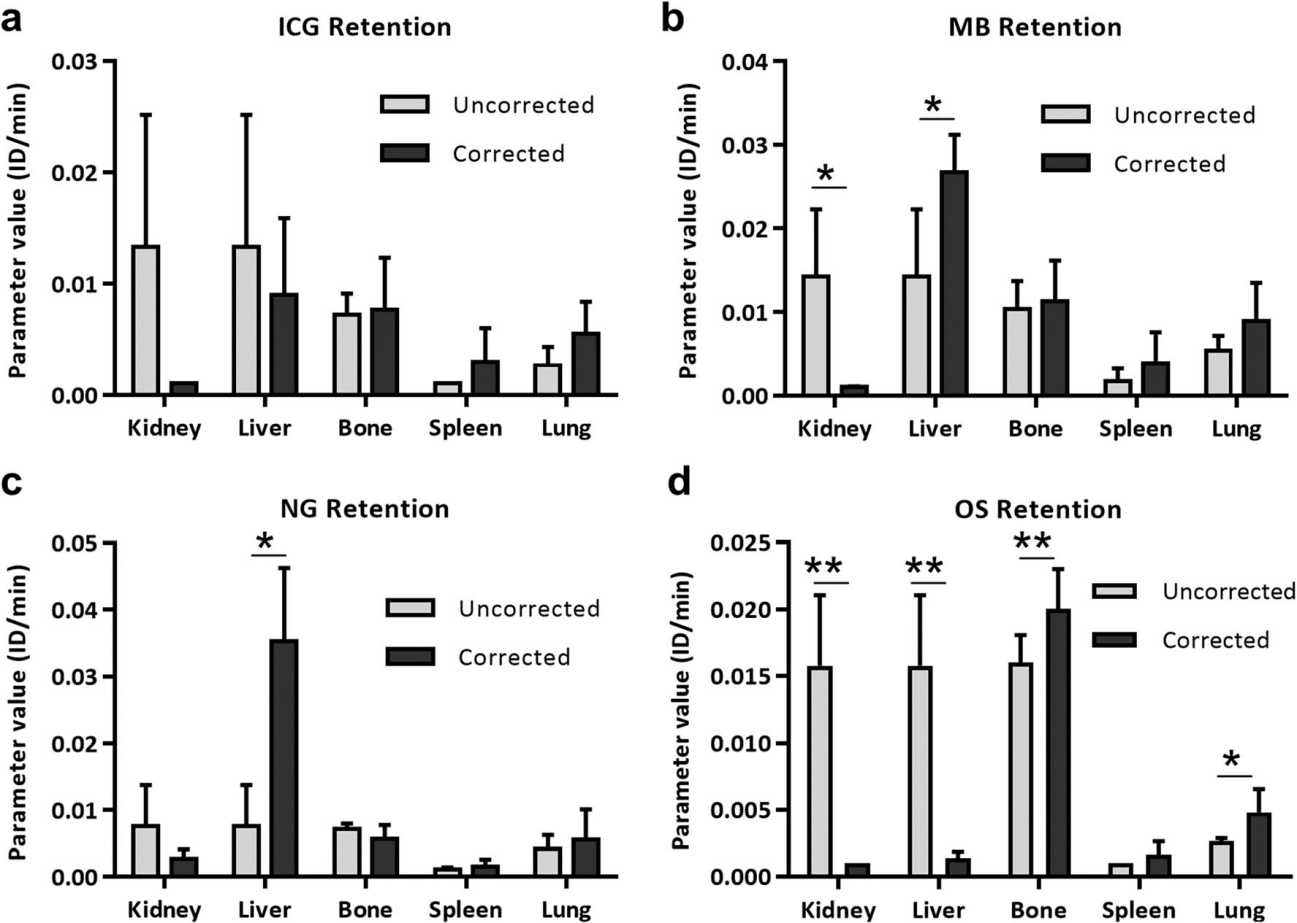
Retention of ICG, MB, NG, and OS in selected organs using the uncorrected and mixing matrix-corrected modeling. **a**–**d** ICG, MB, NG, and OS had negligible renal retention rates when using the corrected modeling compared to the uncorrected one. **b**, **c** MB and NG show a higher hepatic retention rate (P<0.05 and P<0.05, respectively). **d** With correction, OS has a higher bone retention rate (P<0.01), while lower renal and hepatic retention rates (P<0.01 and P<0.01, respectively). The paired two-tailed t-test was used in the comparison of elimination rates between the corrected and uncorrected modeling using μCT-FMT data, n=5.

Figure 6 shows that the usage of the mixing matrix correction lowers the variances of retention parameters. MB and NG were mainly retained in the liver (Fig. 6b, c). With correction, the renal retention rates of ICG, MB, NG, and OS were smaller (MB: P<0.05 and OS: P<0.01, respectively). With the corrected modeling, OS had a significantly higher retention rate in the bone (2.06 ± 0.37 % ID/min) than in the kidneys (0.10 ± 0.00 % ID/min) and liver (0.10 ± 0.00 % ID/min), consistent with the bone-specific property of OS. In addition, the corrected bone retention rate of OS (2.06 ± 0.37 % ID/min) was significantly larger, compared to ICG, MB, and NG (0.95 ± 0.60, 1.17 ± 0.46, and 0.62 ± 0.26 % ID/min respectively). In the corrected modeling, slightly larger spleen and lung retention rates were obtained for all four probes.

The corrected modeling incorporated the retention and elimination sub-compartments in the liver. This incorporation resulted in slightly lower hepatic elimination rates of MB (Fig. 5b, P<0.01) and NG (Fig. 5c, P<0.01) but higher hepatic retention rates of MB (Fig. 6b, P<0.05) and NG (Fig. 6c, P<0.05). Additionally, the renal and hepatic retention rates of OS were significantly smaller using correction (Fig. 6d, P<0.01 and P<0.01, respectively).

## Discussion

Pharmacokinetic modeling is a useful tool to obtain kinetic parameters and facilitates a better understanding of compounds’ kinetic behaviors. Our study aimed to improve the parameter estimation using mixing matrix correction based on longitudinal μCT-FMT data. The mixing matrix “corrected” the measured pharmacokinetic curves, and actual fluorescence intensities of compartments were thus computed. The correction of fluorescence measurements could avoid serious bias in parameter estimation in this study using intravenous injection, especially for compounds distinctively retained in the liver and/or kidneys.

In the simulations, when using the mixing matrix-corrected modeling, stronger linear relationships between the estimated and simulated parameters were achieved, compared to the uncorrected one. The linear relationships of the renal and hepatic retention rates mainly depend on the differentiation of the retention and elimination sub-compartments in the liver and kidneys. Smaller relative errors of estimated parameters are expected since the mixing matrix-corrected modeling minimizes the influence of fluorescence blurring from surrounding organs and the regional blood flow in well-perfused organs, especially in the liver, kidneys, and bone. The high degree of accuracy in parameter estimation indicates that the proposed modeling with mixing matrix correction is robust and reliable.

We also found that sampling with more data points, mostly significantly sufficient measurements in the early pharmacokinetics (particularly within the first hour), could notably suppress the noise and reduce relative errors in parameter estimation. However, the time sampling scheme in the animal experiment is highly limited by the performance of the hybrid μCT-FMT imaging device (13 min per hybrid μCT-FMT scan) [17, 18]. Although the inclusion of μCT imaging provides a more accurate localization of fluorescently labeled compounds [6, 18], it extends the scanning duration and limits the amount of sampling in the early phase. Important pharmacokinetic information is lost because of insufficient measurements from the early stage of probe distribution. The limitation could be overcome by including temporal averaging in the model, or by using a faster device [36]. Such a faster FMT could be developed using CMOS cameras. These relatively inexpensive and fast cameras are smaller, and multiple cameras could be used, e.g., one for the excitation and one for the emission channel. Multiple lasers could be used and operated in interleaved mode, requiring less physical movement.

In the *in vivo* results, the estimated parameters are physiologically meaningful, consistent with the expected elimination and retention properties of these four compounds [18]. ICG and NG had the highest hepatic elimination rates, while OS had the highest renal elimination rate. The hepatic retention was highest for MB and NG, while bone retention was highest for OS.

For the assessment of compound elimination in the *in vivo* data analysis, a minor but noteworthy change is that, for OS elimination, the renal elimination rate is slightly larger than the hepatic elimination rate using the mixing matrix-corrected modeling. This is physiologically meaningful since probes with a hydrodynamic size below the glomerular filtration threshold (<8nm) and positively charged typically undergo substantial renal clearance [29, 31].

The retention rates of compounds were significantly different when using the mixing matrix correction. The hepatic retention rates of MB and NG were considerably larger than the renal retention rates, which is consistent with previous studies [29, 30]. The prolonged retention of these compounds is caused by the rapid uptake but relatively slow excretion by the liver. The bone retention rate of OS was significantly larger than ICG, MB, and NG, consistent with OS’s bone-specific property. Additionally, the spleen and lung retention rates of ICG, MB, and NG are slightly larger. This is reasonable because of the uptake by the mononuclear phagocyte system in these organs [29, 31, 37, 38]. However, the overestimation of the lung retention rate for OS might be a result of the fluorescence spillover in the lung from the neighboring bone tissues [18, 39].

Besides, the pharmacokinetic properties of the fluorescently labeled compounds, i.e., MB and NG, might be influenced by numerous aspects, such as the charge, size, shape surface modifications, solubility, and affinity to plasma proteins or specific receptors [18]. The proposed kinetic modeling can be used to investigate the impact of fluorescence labeling on pharmacokinetic properties, e.g., by comparing compounds with different fluorescent labeling methods.

In this study, some modifications were adopted in the pharmacokinetic modeling, based on the previously presented compartmental modeling [25, 40]. The proposed whole-body modeling incorporates the main elimination routes and major retention sites, which comprise the complete biodistribution of compounds inside the body. The bone targeting property of OS was observed in the animal results, compared to ICG, MB, and NG. This suggests that our proposed modeling can be applied to other fluorescent compounds targeting specific cells and tissues, e.g., a tumor-targeting fluorescent probe.

In this model, the retention sites are simplified only as trapped compartments, where the transfer of compounds between compartments is irreversible. The irreversible retention is physiologically meaningful and suitable, representing the trap of macromolecules in the liver, such as albumin-binding ICG, MB, and NG [29, 31, 37, 38]. However, in some whole-body modeling studies, especially in PET using small radiolabeled compounds with reversible binding to specific organs, the transfer between the blood and tissue compartments is reversible [41, 42]. Besides, limited by the image quality and the temporal resolution, a good blood signal curve was not available in the μCT-FMT kinetic modeling, especially in the early phase of the pharmacokinetic process. In contrast, with high temporal resolution (~seconds), PET kinetic modeling strongly depends on the “arterial input function” and good image quality. This transfer property and the “arterial input function” should be reconsidered to extrapolate the model to PET kinetic data in future studies. Furthermore, considering the complexity of the hepatobiliary elimination and the limited resolution of FMT, the liver is regarded as an isolated organ, whereas 80 % of the hepatic blood supply is from the spleen, gut, and pancreas [43, 44]. These simplifications of the hepatic elimination route need to be reconsidered in animal studies, especially using oral administration.

In the hybrid μCT-FMT imaging, anesthetized mice were imaged to obtain pharmacokinetic curves. Motion artifacts caused by breathing and cardiac movements contribute to fluorescence blurriness and result in inaccurate biodistribution data. Therefore, a mouse bed was applied in the experiments to restrain mice’s motion and limit the impact on fluorescence measurement [17], though, in future *in vivo* studies, the remaining motion artifact problems could be considered to improve the pharmacokinetic analysis.

Despite these limitations, we have demonstrated that the proposed whole-body modeling can improve the parameter estimation with a significantly higher degree of accuracy by involving a mixing matrix. With the new approach, the differences in retention sites can be shown notably more explicit using μCT-FMT animal data. This indicates that the corrected modeling might be a promising tool to assess the pharmacokinetics of organ-specific compounds using *in vivo* data.
